# The Trade in Medicinal Animals in Northeastern Brazil

**DOI:** 10.1155/2012/126938

**Published:** 2011-12-14

**Authors:** Felipe Silva Ferreira, Ulysses Paulino Albuquerque, Henrique Douglas Melo Coutinho, Waltécio de Oliveira Almeida, Rômulo Romeu da Nóbrega Alves

**Affiliations:** ^1^Programa de Pós-Graduação em Ciências Biológicas, Universidade Federal da Paraíba, 58051-900 João Pessoa, PB, Brazil; ^2^Laboratório de Etnobotânica Aplicada, Área de Botânica, Departamento de Biologia, Universidade Federal Rural do Pernambuco, 52171-900 Recife, PE, Brazil; ^3^Departamento de Química Biológica, Universidade Regional do Cariri, 63105-000 Crato, CE, Brazil; ^4^Departamento de Biologia, Universidade Estadual da Paraíba, 58429-500 João Pessoa, PB, Brazil

## Abstract

Over the centuries, a significant part of the Brazilian fauna is widely sold, more specifically in retail stores or street markets. The objective was to characterize the sale of medicinal animals in five large northeast cities. Information about the sale of zootherapeutic items was obtained in the cities of Aracaju-SE, Fortaleza-CE, Maceio-AL, Recife-PE, and Salvador-BA. A total of 68 animal species were sold for medicinal purposes in the cities studied; these are the first results on the use and sale of zootherapeutics in the markets of Aracaju, Fortaleza, and Salvador and first recorded on the medicinal use of the *Achatina fulica, Trachycardium muricatum, Philodryas olfersii, Desmodus rotundus,* and *Leptodactylus vastus*. Knowledge of the fauna utilized popular medicine is indispensable for conservation, demonstrating that research on this subject is necessary to determine appropriate practices for the management of the fauna.

## 1. Introduction

Brazil has a rich diversity of animal species, which, along with its extensive cultural diversity, is reflected in a complex knowledge of the uses of faunistic resources [1–3]. Over the centuries, a significant part of the fauna has been utilized for alternative therapeutic agents by different people of the country [1, 4], a practice that has been perpetuated since colonial times and currently is widely spread among rural and urban communities in various regions of Brazil [5, 6]. In the cities, zootherapeutic products are widely sold, more specifically in retail stores or street markets as noted by recent ethnozoological studies [1, 4, 7–12].

Studies carried out in stores and street markets for the purpose of evaluating the commerce of medicinal animals are scarce [13]. Albuquerque et al. [14] affirm that these outlets can, on a small scale, represent the biodiversity of a region, making it possible to identify areas of extensive exploitation, which can provide information to help monitor the regional biodiversity. From an ecological perspective, the sale of plants and animals at these locations makes them important, since the demand for these resources can have direct and indirect implications on the diversity exploited. Thus, as pointed out by Almeida and Albuquerque [7], the information obtained in these commercial centers can be utilized for the formulation of rational strategies in the commercialization and use of these resources.

Considering that various animal species sold for medicinal use are on lists of threatened species [15], the ecological implications associated with this modality of exploitation of fauna are evident. Whiting et al. [16] noted that biologists and ecologists have neglected information about traditional commerce in devising strategies for the conservation of the species utilized for the production of traditional remedies. Therefore, ethnozoological studies are important, because they provide information about the species utilized for traditional purposes (medicinal, religious, food, etc.), which can contribute to the development of actions that allow the maintenance of faunistic resources [7, 13, 17]. 

In Northeast Brazil, the commerce of zootherapeutic products is common. Recent studies conducted in 10 cities in the region [5, 12, 18–22] demonstrated the existence of intense use and commercialization routes of animals for medicinal purposes among these cities. However, there are still gaps concerning the richness of traded species, which makes it difficult to evaluate the magnitude and impact of this commerce on natural populations of the animals involved, as well as potential implications of such uses for the public health of local users.

Despite the existence of information on the sale of animals for medicinal purposes in some important cities of Northeast Brazil, such as Aracaju-SE, Fortaleza-CE, and Salvador-BA, there are no published data available. For the cities of Maceio-AL and Recife-PE, although the commerce of medicinal fauna has been previously investigated, the list of species presented does not include all taxonomic groups but shows few animals identified at the species level.

The objective of the present study was to characterize the sale of medicinal animals in five large northeast cities. More specifically, the work aimed to (i) list which animals are sold for medicinal purposes in the capitals of the states of Sergipe (Aracaju), Ceará (Fortaleza), Alagoas (Maceió), Pernambuco (Recife), and Bahia (Salvador); (ii) evaluate the versatility of the animal species by calculating the relative importance; (iii) test the idea of utilitarian redundancy; (iv) compare the degree of similarity between the localities sampled; (v) estimate the richness and diversity of species traded; and based on this information, discuss aspects related to conservation and public health associated with the medicinal fauna commerce in Brazil.

## 2. Materials and Methods

### 2.1. Area of Study

**Figure 1:**
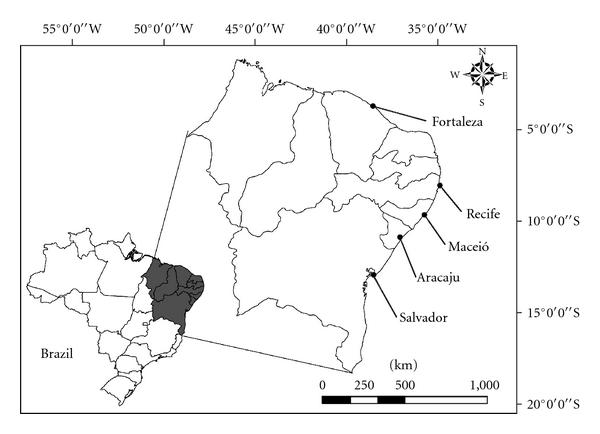
Map locating the cities studied in Northeastern Brazil.

Information about the use and sale of zootherapeutic products was obtained in the cities of Aracaju-SE, Fortaleza-CE, Maceio-AL, Recife-PE, and Salvador-BA (Figure 1). In Maceio and Recife, prior studies on the sale of zootherapeutic products have been carried out [5, 22], where the first involved only one taxonomic group (reptiles) and both works did not show information on the number citations among the vendors, which prompted us to include these cities in our research.

### 2.2. Procedures

Field work was undertaken during the period from January to November of 2010, in public markets of Aracaju (Mercado Central), Fortaleza (Mercado Central, São Sebastião, and Praia do Futuro), Maceió (Mercado da Produção), Recife (Mercado São José, Afogados, Água Fria, Encruzilhada, and Casa Amarela), and Salvador (Feira de São Joaquim, Sete Portas, and Itapuã).

To obtain the information we interviewed 102 (65 men and 37 women) merchants about the use and commercialization of medicinal animals, being 12 in the Aracaju city (11 men and 1 woman), 29 in Fortaleza (17 men and 12 women), 17 in Maceió (7 men and 10 women), 21 in Recife (16 men and 5 women), and 23 in Salvador (14 men and 9 women).

Sampling was nonrandom intentional, in which the interviewees were predefined [23], composed only of people who actually sold zootherapeutic products. Semistructured questionnaires were used, complemented by free interviews and informal conversations. The questionnaires contained questions on the animal species used for medicinal purposes, their respective uses, preparations, and parts utilized.

To respect intellectual property rights, we adopted the following protocol in the field: before the survey, we introduced ourselves, explained the nature and objectives of our research, and asked the respondents for permission to record the information. The ethical approval for the study was obtained from the Ethics Committee of Universidade Federal da Paraíba (Protocol: CEP/HULW no. 065/10).

Vernacular names of species were recorded as quoted during the interviews. Zoological material was identified with the aid of specialists, through examination of voucher specimens donated by the interviewees or purchased at the surveyed markets, and through photographs taken during interviews of the animal species or their parts. Whenever necessary, these procedures were supplemented by checking vernacular names provided by traders against the scientific names, with the aid of taxonomists familiar with the study areas.

### 2.3. Data Analysis

The ailments treated by zootherapeutics were grouped into categories based on the model used by the “Centro Brasileiro de Classificação de Doenças” (Brazilian Center for the Classification of Diseases) [24].

To estimate the level of agreement between interviewees over which animals to use for each category, we calculated the informant consensus factor (ICF), adapted from Heinrich et al. [25] that looks at the variability of animals used for each treatment, and therefore the consensus between practitioners. This factor estimates the relationship between the “number of use reports in each category (*nar*) minus the number of taxa used (*na*)” and the “number of use reports in each category minus 1.” ICF is thus calculated using the following formula:

(1) $$\text{ICF}=\;\frac{nar\;-\;na}{nar\;-\;1}.$$

The product of this factor ranges from 0 to 1. A high value (close to 1) indicates a high consensus, where relatively few taxa (usually species) are used by a large proportion of people, while a low value indicates that the informants disagree on the taxa to be used for treating a particular illness.

### 2.4. Relative Importance (RI)

The relative importance (RI) of the species cited was calculated (adapted from Bennett and Prance [26]). Relative importance was calculated according to the following formula, with “2,” being the highest possible value, indicating the most versatile species. The most versatile species are those that have the greatest number of medicinal properties: RI = NCS + NP, where NCS (number of body systems) is the number of body systems treated by a given species (NCSS) divided by the total number of body systems treated by the most versatile species (NCSSV). The number of properties (NP) is obtained by the relationship between the number of properties attributed to a species (NPS) divided by the total number of properties attributed to the most versatile species (NPSV).

### 2.5. Utilitarian Redundancy of the Diseases and/or Symptoms

Utilitarian redundancy of zootherapeutic products was tested according to the model adapted from Albuquerque and Oliveira [27]. According to these authors, the idea of utilitarian redundancy is based on the theory of ecological redundancy (this theory indicates that all species present specific functions in the ecosystem, but some can show similar functions, minimizing damages in the ecosystem due the extinction) (see [28, 29]). Therefore, the notion of functional redundancy relies on the presumption that some species are utilized for the treatment of more than one disease and/or symptom, such that the inclusion of more than one species within a disease category can be a mechanism of reducing the impact on the animals sold for medicinal purposes.

To evaluate this hypothesis, each disease and/or symptom was categorized according to the level of redundancy of the species used: highly redundant (≥15% of the number of species utilized), redundant (15% < the number of species ≥5%), and not very redundant (<5% of the species). In order to evaluate the idea of utilitarian redundancy in a possibly better manner, the diseases and/or symptoms were not reclassified, and thus the names cited by the informants were kept.

### 2.6. Coefficient of Similarity

The composition of the species cited was compared between the cities studied by means of the similarity index based on data of multiple incidence. The similarity between the localities was estimated using the distance coefficient of Bray-Curtis [30]. The similarity matrix was constructed and grouping analysis performed in the past program [31].

### 2.7. Estimate of Species Richness

Initially, incidence data (presence or absence) of the species in the markets of Aracaju, Fortaleza, Maceio, Recife, and Salvador were used to estimate the richness of medicinal species sold in each city. The term richness of species refers to the number of species living in the determined area [32]. However, due to the difficulty to access the total number of species, indexes of estimated richness are important tools to identify the most probable number of species living on the ecosystem, community, or, as our work, on the public markets. Species richness was calculated utilizing the estimators CHAO 2, ICE, Jackknife 1, and Jackknife 2 (see [30]), with the program Estimate S 8.2.0 [33]. These indices have been utilized in ethnobotanical and ethnozoological studies [16, 34].

## 3. Results and Discussion

**Figure 2:**
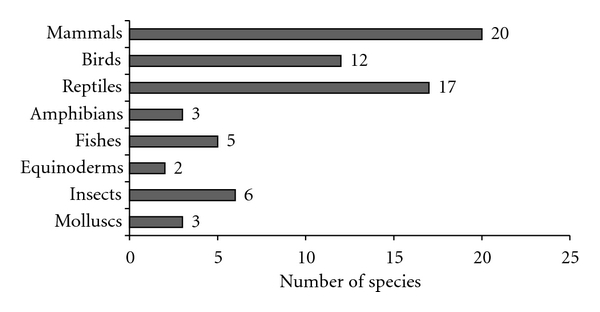
Number of animal species used as remedies per taxonomic category in Northeastern Brazil.

In the cities studied, the trade in animal-based medications was shown to be common practice. A total of 68 animal species, distributed in 47 families, were sold for medicinal purposes in the cities studied (Table 1). The most representative taxa were mammals (20), reptiles (17), and birds (12) (see Figure 2). These are the first results on the use and sale of animals for traditional medicine in the markets of Aracaju, Fortaleza, and Salvador, where the commercialization of respectively 19, 28, and 36 species was recorded. For the cities of Recife and Maceio, the number of species recorded was higher than that documented in previous works. In Recife, Silva et al. [5] recorded the commerce of 18 medicinal species, while in Maceió, Freire [22] reported the use of 17 species of reptiles sold for medicinal purposes. In the present study, we recorded 31 and 27 species in Recife and Maceió, respectively.

The increase in the number of species cited for medicinal purposes, sold in the markets of Recife and Maceio may be the result of the structure of the traditional medicine systems of the public markets, because these are open and dynamic systems, which are inclined to increase in species over the years, although with a tendency to maintain the species of greater importance [14]. These authors studied the sale of medicinal plants in the same market in different periods (1995 and 2002) and found an increase of 58 species in a period of seven years (1995 = 78 species and 2002 =136 species), but species of high relative importance continued to be present in the markets evaluated.

Similarly, the data obtained in the present study corroborated the results of Albuquerque et al. [14] for the commerce of medicinal plants. We found an increase in the composition of animal species in the markets of Recife and Maceio, where the sale of species of high relative importance was maintained, such as *Caudisona durissa* and *Tupinambis merianae*, which were recorded in the two periods in which the studies were performed.

In general, the diversity of medicinal species recorded in the present study confirms the importance of the fauna as a therapeutic resource in urban areas, corroborating previous studies that indicated the commercialization of zootherapeutic products as a common activity in various Brazilian cities (see [8, 12, 17–21]). Compared to other studies of markets in Northeast Brazil, the number of medicinal species traded in the cities studied is substantial. In Feira de Santana, BA, for example, a total of 16 animal species were reported being sold for medicinal purposes in the public markets of the city [18], in Santa Cruz do Capibaribe, PE, 37 species [19], in Caruaru, PE, 36 species [20], in the cities of Crato and Juazeiro do Norte 31 species [12], and in the metropolitan region of Natal, 23 species [21].

**Figure 3:**
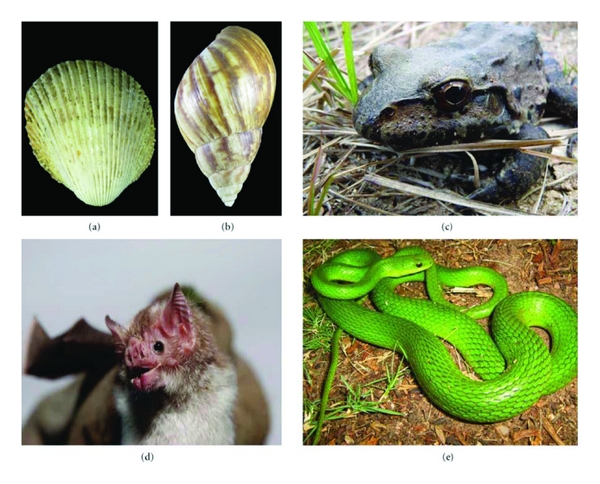
New records of species used in traditional medicine. (a)* Trachycardium muricatum*, (b) *Achatina fulica*, (c) *Leptodactylus vastus*, (d) *Desmodus rotundus*, (e) *Philodryas olfersii *(Photos: (a), (b) Joafrâncio P. Araújo; (c) Hugo Fernandes-Ferreira; (d) Patrício A. da Rocha; (e) Samuel C. Ribeiro).

The majority of the medicinal species sold in the cities studied are the same as those sold and/or utilized in other cities in Northeast Brazil, with the exception of five medicinal species not previously recorded (Figure 3): *Achatina fulica*, *Trachycardium muricatum*, *Philodryas olfersii*, *Desmodus rotundus,* and *Leptodactylus vastus*. Of these species, four were cited only in the Salvador markets (*A. fulica* [cited by five informants], *T. muricatum* [cited by two informants], *P. olfersii* [cited by one informant], and *D. rotundus* [cited by three informants]) and only one in the Fortaleza markets (*L. vastus* [cited by three informants]).

The species of molluscs *A. fulica* and *T. muricatum* are utilized in the treatment of stroke. The fat of *L. vastus* is administered for the treatment of sore throat, cough, asthma, arthritis, and backache. The snake *P. olfersii* is utilized for the treatment of stroke and the bat *D. rotundus* is administered for the treatment of stroke, asthma, and rheumatism.

The species with the highest number of citations were *Apis mellifera* (*n* = 46), *Tupinambis merianae* (*n* = 28), *Hippocampus reidi* (*n* = 27), *Bos taurus* (*n* = 23), *Oreaster reticulatus* (*n* = 20). In other studies on the trade of zootherapeutic products in stores and street markets, these species are also often utilized in the production of traditional remedies [8, 12, 17–21].

 Of the species recorded in the present work, the majority represent wild animals (82.4%). Only 12 species of domestic animals are sold as medicinal products, and they are *Anser anser*, *Anas platyrhynchos*, *Numida meleagris*,* Gallus domesticus*, *Pavo cristatus*, *Ovis aries*, *Capra hircus*, *Bos taurus*, *Bubalus bubalis*, *Canis lupus,* and *Sus scrofa. *These results corroborate the tendency observed in other studies, which have demonstrated that wild animals compose the greater part of the medicinal fauna utilized in popular medicine in Brazil [8, 12, 17–21] and in the world [35–40].

 All animals cited occur in ecosystems close to the cities studied, with the exception of *Electrophorus electricus*. This species occurs in the Northeast Brazil region, only in the state of Maranhão [41]. Thus, the results indicate a tendency of the commercialization of animals that occurs in the proximity of the localities sampled. This demonstrates the importance of the local fauna in supplying the products utilized in the preparation of traditional remedies, which would reduce the costs for the acquisition and commercialization of zootherapeutic products, but this hypothesis needs to be adequately tested. These data corroborate other works conducted in markets in Northeast Brazil [7–9, 12, 17, 42], which also recorded a predominance of the use of animals of the local fauna for trade, showing the importance of the biodiversity of each region as a resource for zootherapy.

**Figure 4:**
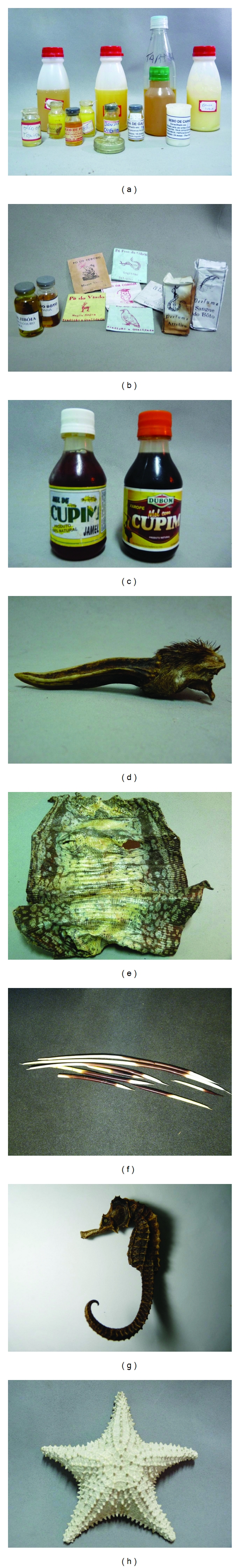
Examples of animal products used as remedies sold in Aracaju-SE, Fortaleza-CE, Maceió-AL, Recife-PE, and Salvador-BA public markets. (a) Body fat; (b) metabolism secretion such as blood, feces, and urine; (c) honey of *Nasutitermes corniger*; (d) horn of *Mazama gouazoubira*; (e) skin of *Tupinambis merianae*; (f) spine of *Coendou prehensilis*; (g) dried Seahorses (*Hippocampus reidi*); (h) dried starfish (*Oreaster reticulatus*). (Photos: Hugo Fernandes-Ferreira).

 Various parts and metabolic secretions of the animals are utilized in the preparation of medications (Figure 4), and they are skin, fat, honey, wax, shell, wings, spines, rattle, blood, feces, horn, feathers, hoof, tibia, cartilage, eye, tail, liver, claw, foot, eggs, bile, and bone. Animals such as *A. fulica, T. muricatum*, *P. olfersii*, *D. rotundus*, *O. reticulates,* and *H. reidi* can be used whole.

Among the products cited by the informants, fat was cited most often, which can be extracted from the following animals: *H. malabaricus*, *E. electricus*, *R. jimi*, *C. mydas*, *T. Merianae,* and *B. constrictor*. The frequent utilization of fat can be attributed to the fact that the main animals utilized are vertebrates, which have a large quantity of fat in their body [19]. Another possible explanation for the marked use of body fat for medicinal purposes can be due its chemical composition. Body fat consists mainly of fatty acids, which have an extensive proven medicinal applicability [43–45], such that this intense use and/or medicinal trade can be the result of the empirical observation of the efficacy of fat by human users of this zootherapeutic product.

The medicinal animals listed in the present study are applied for the treatment of 58 diseases and/or symptoms (Table 2). The categories with the higher values of ICF were diseases of the respiratory tract (0.91), diseases of the musculoskeletal system and connective tissue (0.89), and undefined diseases (0.88). These high values of consensus for these categories were also found in other works carried out in the public markets of North and Northeast Brazil [8, 9, 12, 19, 20].

 A total of 1575 citations of uses for medicinal animals were cataloged (Table 3). The categories with highest number of citations were diseases of the respiratory tract (613 citations; 56 species), diseases of the musculoskeletal system and connective tissue (269 citations; 29 species), and undefined diseases (259 citations; 38 species). The diseases with highest number of citations were asthma (226 citations; 14.3%), sore throat (158 citations; 10.1%), and cough (111 citations; 7.1%). Other works carried out in the Northeast also indicate that these diseases are widely treated with medicinal animals [7–9, 12, 19, 20, 40].

Even with the high number of citations to illnesses treated with animal products commercialized in Brazil, there are few laboratory studies testing its efficacy. Ferreira et al. [45] indicate that the body fat of *Boa constrictor* does not present a clinically relevant bacterial activity, but when combined with antibiotics, the fat demonstrated a significant synergistic activity. Similar results are reported to the decoction of the lizard *Tropidurus hispidus* and the termite *Nasutitermes corniger* (see [46–48]). But et al. [49] report the antifever activity of the preparations using the horn of *Bos taurus*. Murari et al. [50] and Ferreira et al. [44] report that extracts of *Pavo cristatus* and the body fat of *Tupinambis merianae *demonstrated anti-inflammatory activity. Tempone et al. [51] showed that steroids from the skin of *Rhinella jimi* are active against leishmaniasis and trypanosomiasis. Besides the high number of animal species commercialized with medicinal uses in Brazil, studies about the improved biological activity of theses products are still preliminary and insufficient. So, the development of more studies is necessary to understand, evaluate, and validate the traditional and medicinal knowledge associated with the use of animal products.

Zootherapeutic remedies can be prepared in the following ways: (a) whole animals or body parts are utilized by maceration, where the resultant powder is ingested in the form of teas or together with food, and (b) body secretions and fat are administered as an ointment or ingested.

According to the informants, 60 species (88.2%) are of multiple uses, that is, they are administered in the treatment of more than one disease and/or symptom. The most versatile species, that is, with the highest RI values are *Sotalia guianensis *(1.90), *Trichechus manatus* (1.87), *Caudisona durissa* (1.70), and *Tupinambis merianae *(1.67). Alves et al. [20] and Almeida and Albuquerque [7] also cite *C. durissa* and *T. merianae* as versatile medicinal species in other studies carried out on the commerce of zootherapeutic products.

In contrast, the results obtained in this study show that the same disease and/or symptom can be treated with more than one animal species, demonstrating utilitarian redundancy as proposed by Albuquerque and Oliveira [27]. Among the diseases treated with zootherapeutic products in the cities sampled in the present work, 19 are “highly redundant,” 23 are “redundant,” and 15 are “not very redundant.” Diseases such as asthma, sore throat, rheumatism, and cough are examples of categories “highly redundant.”

**Figure 5:**
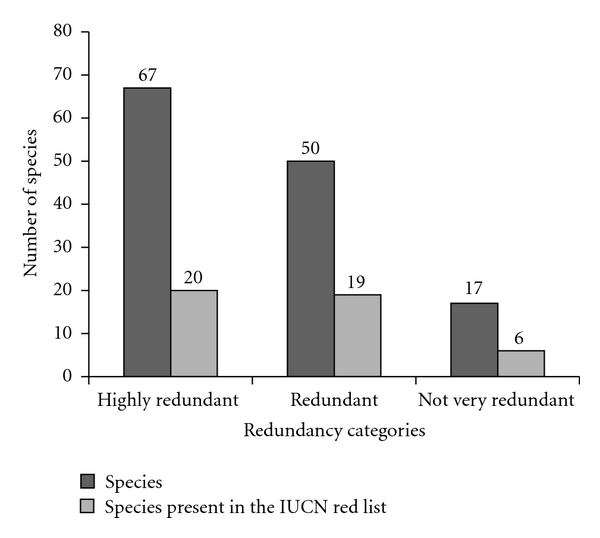
Distribution of the number of species cited per utilitarian redundancy category according to informants of the Aracaju-SE, Fortaleza-CE, Maceió-AL, Recife-PE, and Salvador-BA.

As shown in Figure 5, many species are included in the categories “highly redundant” and “redundant” (67 and 50, resp.), while few species are included in the category “not very redundant” (17 species). Based on the model of utilitarian redundancy, the pressure probably caused in the commercialized species in the markets evaluated is small, because the majority of the species are listed in the categories “highly redundant” and “redundant,” where they also have various alternative therapeutic uses. In general, 23 species are on the red list of the IUCN [52], where six are in the category data deficient, 12 in the category low risk, and one in each of the following categories: near threatened, vulnerable, endangered, and critically endangered. In addition, the proportion of species considered threatened did not differ between the redundancy categories analyzed (chi squared = 0.435; *P* > 0.05).

According to Albuquerque and Oliveira [27], the model of utilitarian redundancy suggests that the inclusion of species in the same therapeutic category can lead to reducing the pressure on the medicinal use of animals, such that species included in redundant categories would have options of equivalent products in other species. In this context, the species included in the category “not very redundant” should be prioritized in the development of conservation strategies, because there would not be equivalent species for medicinal use. However, the species in the more concerning categories of the IUCN red list (*Rhea Americana*: near threatened; *Mazama gouazoubira*: vulnerable; *Chelonia mydas*: endangered; *Trichechus manatus*: critically endangered) were not reported as medicines to the treatment of the “not very redundant” diseases, reinforcing our point of view that the commercialization of animals to medicinal uses do not cause a great pressure over the wild livestocks of animals.

However, this interpretation, about prioritized in the development of conservation strategies, should be taken with caution, since Albuquerque and Oliveira [27] emphasized that even in a redundant category, if there are species that have a greater local preference, the pressure of use would certainly be shifted to them. In addition, the idea of redundancy can be applied to the resilience of the local medical system, that is, highly redundant categories would be, in principle, more resilient than those not very redundant.

**Figure 6:**
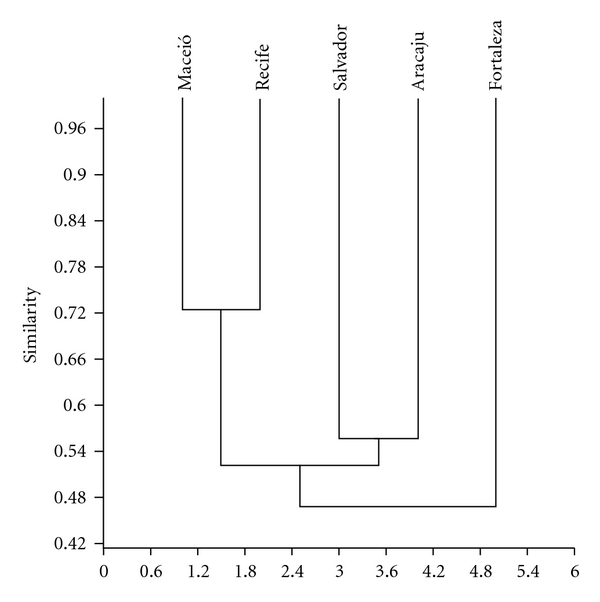
Cluster analysis of the species cited in the surveyed cities. (Correlation coefficient: *R* = 0.93).

In relation to the similarity of the cities sampled (Aracaju, Fortaleza, Maceio, Recife, and Salvador), grouping analysis showed that the greater degree of similarity observed was between Maceio and Recife (Figure 6). In the grouping analysis, we can see that the cities close to each other showed greater similarity with regard to the animal species commercialized. Based on these results, these groupings can likely be a reflection of the presence of similar ecosystems in the cities sampled or the presence of more intense commercial routes of zootherapeutic products between closer cities.

**Figure 7:**
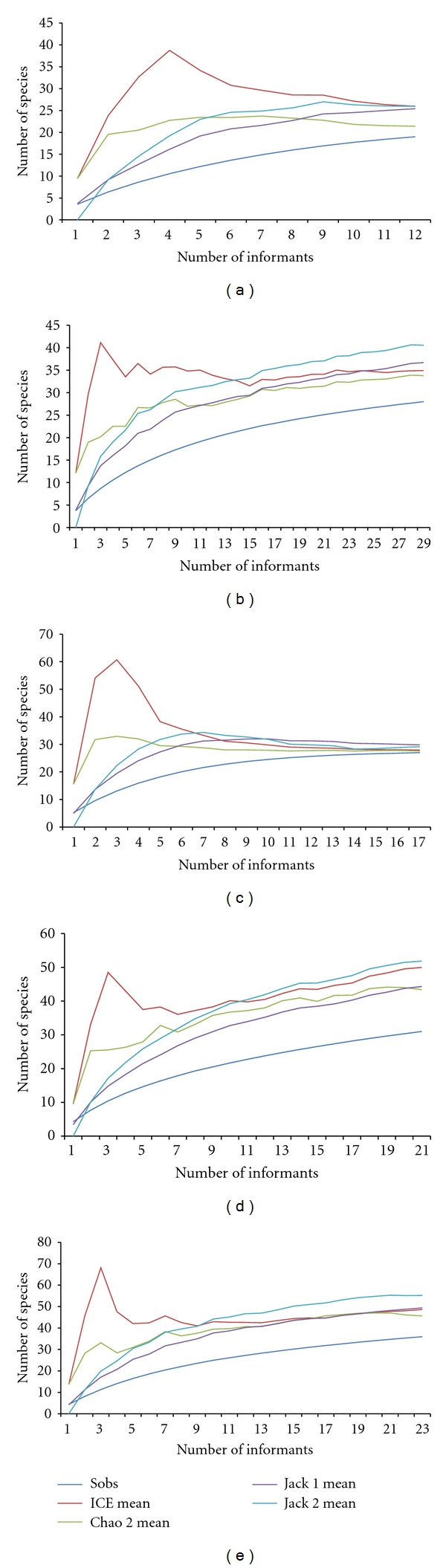
Graphs showing the values obtained with the richness estimators species assessed for each market. (a) Aracaju; (b) Fortaleza; (c) Maceió; (d) Recife; (e) Salvador.

Data on the commerce of medicinal animals are difficult to obtain, because many of the venders do not admit that they utilize or sell products originating from the fauna knowing that it can be illegal [17]. Therefore, the use of estimators of species richness represents an important tool. Our analyses demonstrate this, pointing out that the number of species commercialized tends to be greater than that recorded (Table 4 and Figure 7).

It was observed that for Aracaju, Fortaleza, Recife, and Salvador the estimators indicated the existence of more traded species than that recorded in the present work. In accordance with the study by Whiting et al. [16], in the market in Faraday, South Africa, the richness estimator Jack 2 was better for use in studies on zootherapeutic products. According to these authors, Jack 2 yielded values closer to the number of species observed. However, based on our results obtained with the estimators ICE and CHAO 2 for the data on the sale of animals in Maceio (see Table 4), we can infer that these two estimators are also reliable, because we obtained values close to those obtained through informants in the markets of Maceió.

The values obtained with richness estimators show that the richness of species sold for traditional medicines in Northeast Brazil is high. However, the scarcity of studies on zootherapy in the country, as all over the world, has led to the importance of the zootherapeutic resources being underestimated in the country.

Estimates of species richness were utilized in ethnobiological studies conducted by Begossi [34]. In the case of research on the use and/or commerce of animals or plants, this tool has been little exploited. According to Williams et al. [53], the use of indices of species richness and diversity in ethnobiological research can serve to (i) evaluate the amount of biodiversity human populations exploit; (ii) make it possible to compare communities (or markets) using quantitative data; (iii) infer the minimal number of species necessary for the maintenance of the uses by traditional communities. In summary, the use of these indices open new perspectives for ethnozoological studies, since they can provide estimates on the richness and diversity of animal species utilized, especially considering the difficulty in obtaining information about the trade of wild animals, which are generally carried out in a clandestine manner.

 In the present work, we recorded at least 68 species sold in the cities studied. Of these species, 23 (33.8%) are on the red list of threatened species [52]. The categories in which these species are included are from data deficient up to endangered. Even for some species not considered in high risk categories, the medicinal use and trade are cited as one of the causes of threat and/or population decline for only one species (*H. reidi*). For the majority of commercialized species, however, medicinal trade is not considered a form of threat, although it represents an additional pressure, which should be monitored, especially for species that are extensively exploited.

It is important to point out that the medicinal use of animals cannot be considered the only threat to the conservation of the species utilized for these purposes. Some authors [54] point out that the remedies based on animals are mainly formed of subproducts that do not serve other purposes other than medicinal, and therefore, the true reason for hunting them may not be for medicinal use, such in the case of food.

 Understanding the aspects that involved the commerce of medicinal animals is important for the formulation of management plans for sustainable use of medicinal animal species [3]. Some works on the sale of medicinal animals indicate a concern with respect to the maintenance of these faunistic resources, taking into consideration that in Brazil the sale of medicinal animals in stores and street markets is not monitored [13]. 

In other countries, some published works indicate that the trade of fauna for various purposes, including medicinal, is one of the main causes of threats to wild populations. Servheen [55] pointed out that 14 species of bears, on the IUCN red list, are traded in and outside of China for medicinal use without the monitoring of the number of individuals sold. According to Lee [56], the use of rhinoceros horn in traditional medicine has been indicated as one of the main causes for the population decline of these animals. Alves et al. [3], providing an overview of the global use of primates in traditional folk medicines, noted that >100 species were traded for this purpose, and as noted by Ahmed [57] unchecked exploitation is leading to decreasing populations of primates utilized in traditional medicine in India. In Indonesia, Lee et al. [58] pointed out that the sale of mammals for various purposes, including medicinal, is one of the causes for declines in mammal populations. Athiyaman [59] reported that species of tigers are among the animals most endangered due to its trade for medicinal purposes without monitoring. Zhang et al. [60] stated that in China one of the major causes for the decline in species is illegal trade for food, craftwork, and medicinal purposes.

In Brazil, there is still no information that indicates the decline of species due to traditional medicine trade, although this activity has been indicated as one of the causes of a population decline of *H. reidi* [61, 62]. Although the intense commercialization of animals for medicinal purposes does not represent a significant impact for most of the species, such uses should be considered in strategies of management and conservation, particularly for those medicinal animals that are exploited more and on the list of threatened species [15].

Considering that animals represent an important source of remedies used in traditional medicines, zootherapy has become extremely relevant from a conservationist viewpoint [3]. In all the world, populations of various species have been utilized and the demand created by traditional medicine is probably one the causes of overexploitation found for some species of large mammals [1, 55, 57, 63].

According to Alves and Rosa [1], the ecological aspects associated with zootherapy represent one of the main reasons for studying the use of animals for medicinal purposes. However, it has not been possible to evaluate the magnitude of the impact of the medicinal use of the fauna, since the ways in which animals are used vary greatly [41], and the zootherapeutic products can be obtained indirectly from hunting for other purposes [11, 64]. Therefore, medicinal demand should be considered within a greater context of use of the fauna. The frequent commercialization of zootherapeutic products derived from particular species and their respective conservation status demonstrate that some animals deserve special attention.

In general, the use and commercialization of medicinal animals in Northeast Brazil is a reality consisting of an alternative for the treatment of various diseases, as well as representing an important source of income for various people. Knowledge of the fauna utilized popular medicine is indispensable for conservation, demonstrating that research on this subject is necessary to determine appropriate practices for the management of the fauna, thereby allowing the maintenance of the medicinal resources utilized and of the medicinal knowledge associated with these resources.

## Figures and Tables

**Table 1:** Animal species commercialized for medicinal purposes in the municipalities of Aracaju-SE, Fortaleza-CE, Maceió-AL, Recife-PE, and Salvador-BA.

Family/species/local name	Part used	Disease (or illness)	RI total	Number of citations per city/RI per city				
				Aracaju	Fortaleza	Maceió	Recife	Salvador
*Molluscs*								
Achatinidae								
*Achatina fulica *(Ferussac, 1821), giant east African snail, caramujo gigante africano	Shell	Stroke	0.12	—	—	—	—	5/0.14
Cardiidae								
*Trachycardium muricatum* (Linnaeus, 1758), yellow pricklycockle, rala-côco	Shell	Stroke	0.12	—	—	—	—	2/0.14
Strombidae								
*Strombus pugilis* Linnaeus, 1758, west Indian fighting conch, estrombo-lutador- das-Índias-Ocidentais	Shell	Stroke and “simpatias”	0.23	2/0.56	—	—	—	1/0.14

*Insects*								
Apidae								
*Apis mellifera* (Linnaeus, 1758), honey bee, abelha italiana	Honey and wax	Bronchitis, cough, flu, nasal congestion, asthma, sore throat, stomach ache, diarrhea, and cracks in the feet	0.62	4/0.78	22/1.02	4/0.32	10/0.34	6/0.36
*Melipona subnitida *(Ducke, 1910), stingless bee, jandaíra	Honey and wax	Sore throat, cough, and diarrhea	0.18	—	4/0.55	—	—	—
*Melipona scutellaris *Latreille, 1811, stingless bee, uruçu	Honey and wax	Asthma, cough, flu, nasal congestion, earache, stomach ache, diarrhea, and gastritis	0.59	—	—	4/1.09	1/0.18	—
*Partamona cupira *(Smith, 1863), stingless bee, abelha cupira	Honey and wax	Sore throat, flu, cough, nasal congestion, and diarrhea	0.33	—	2/0.55	2/0.43	1/0.34	—
Blattidae								
*Periplaneta americana* (Linnaeus, 1758), cockroach, barata	Wings and whole animal	Asthma and earache	0.23	2/0.56	—	—	—	—
Termitidae								
*Nasutitermes corniger* (Motschulsky, 1855), térmite, cupim de aroeira	Whole animal	Asthma, cough, flu, and sore throat	0.21	—	4/0.45	—	—	—

*Equinoderms*								
Echinasteridae								
*Echinaster echinophorus* (Lamarck, 1816), starfish, estrela do mar	Whole animal	Asthma, flu, stroke, “simpatias,” erysipelas, cancer, and thrombosis	0.64	7/0.83	—	3/0.44	—	6/0.60
Oreasteridae								
*Oreaster reticulatus* (Linnaeus 1758), starfish, estrela do mar	Whole animal	Asthma, stroke, erysipelas, cancer, “simpatias,” and evil eyes	0.61	6/0.83	1/0.24	3/0.54	6/0.59	4/0.56

*Fishes*								
Erythrinidae								
*Hoplias malabaricus* (Bloch, 1794), trhaira, traíra	Fat	Earache, healing, strain, sore throat, cough, asthma, swelling, urinary infection, and infections	0.79	2/1.39	4/1.17	—	—	—
Gymnotidae								
*Electrophorus electricus*, (Linnaeus, 1766), electric eel, peixe-elétrico, LR	Fat	Rheumatism, osteoporosis, crack in the feet, pains, inflammation, sore throat, asthma, cough, sinusitis, flu, swelling, itch, wound, earache, toothache, burns, muscular pain, bruises, strain, headache, joint pain, erysipelas, snake bites, skin diseases, healing, and boils	1.51	1/0.67	4/1.79	2/1.41	1/0.64	3/1.26
Syngnathidae								
*Hippocampus reidi* (Ginsburg, 1933), longsnout seahorse, cavalo-marinho, DD	Whole animal	Asthma, flu, stroke, erysipelas, toothache, circulatory problems, and thrombosis	0.64	4/0.39	4/0.24	4/0.54	7/0.28	9/0.78
Unidentified family								
Shark, tubarão	Cartilage	Rheumatism	0.12	—	1/0.24	—	—	—
								
Unidentified family								
Stingray, Raia	Fat	Sore throat, pains, rheumatism, burns, asthma, and infections	0.53	—	1/1.26	—	—	—

*Amphibians*								
Bufonidae								
*Rhinella jimi* (Stevaux, 2002), cururu toad, sapo cururu, LR	Skin and fat	Sore throat, asthma, flu, cough, rheumatism, inflammation, backache, osteoporosis, arthrosis, arthritis, strain, diarrhea, “simpatias,” toothache, cancer, infections, and earache	1.13	1/0.67	7/1.45	—	1/0.94	1/0.18
Leptodactylidae								
*Leptodactylus labyrinthicus* (Spix, 1824), South American pepper frog, rã-pimenta, LR	Fat	Sore throat, cough, and asthma	0.18	—	2/0.38	—	—	—
*Leptodactylus vastus* Lutz, 1930, South American pepper frog, rã-pimenta, LR	Fat	Sore throat, cough, asthma, arthritis, and backache	0.33	—	3/0.69	—	—	—

*Reptiles*								
Chelidae								
Tortoise, cágado_Fortaleza	Fat	Rheumatism, cracks in the feet, arthrosis, backache, sore throat, and swelling	0.53	—	2/1.10	—	—	—
Tortoise, cágado_Recife	Carapace	Asthma, burns, and rheumatism	0.35	—	—	—	1/0.53	—
Cheloniidae								
*Chelonia mydas* (Linnaeus, 1758), green turtle, tartaruga verde, EM	Fat and carapace	Sore throat, rheumatism, swelling, inflammation, burns, healing, cough, asthma, arthritis, backache, cancer, backache, stroke, thrombosis, erysipelas, stomach ache, and infections	1.18	—	4/1.38	—	—	1/0.92
Testudinidae								
*Chelonoidis* sp, tortoise, jabuti	Fat	Sore throat, cough, asthma, earache, backache, and inflammations	0.53	—	1/1.17	—	—	—
Iguanidae								
*Iguana iguana* (Linnaeus, 1758), common green iguana, camaleão, DD	Fat and tail	Earache, deafness, sore throat, inflammations, swelling, wounds, burns, and acne	0.67	—	—	3/0.71	1/1.05	—
Teiidae								
*Cnemidophorus ocellifer* (Spix, 1825), spix's whiptail, calango	Whole animal	Stroke, thrombosis, cancer, and hemorrhoids	0.30	—	—	2/0.71	—	—
*Tupinambis merianae* (Duméril and Bibron, 1839), teju lizard, téju, LR	Fat, skin, and tail	Sore throat, pneumonia, sinusitis, flu, bronchitis, asthma, cough, rheumatism, swelling, strain, inflammations, earache, deafness, backache, arthrosis, arthritis, burns, osteoporosis, healing, muscular pain, cracks in the feet, toothache, headache, itch, lung problems, infections, pains, injuries, gastritis, snake bites, and skin diseases	1.67	1/2.00	12/1.83	3/1.79	8/1.82	5/1.66
Boidae								
*Boa constrictor* (Linnaeus, 1758), boa, jibóia, DD	Skin, fat, bone, and feces	Sore throat, cough, toothache, rheumatism, inflammations, asthma, flu, arthrosis, osteoporosis, cracks in the feet pains, arthritis, backache, healing, “simpatias,” swelling, bruises, cancer, tuberculosis, pneumonia, edemas, and stomach ache	1.29	2/1.67	8/1.60	3/1.52	—	2/0.86
*Epicrates cenchria* (Linnaeus, 1758), rainbow boa, salamanta, DD	Fat	Sore throat, rheumatism, swelling, backache, arthrosis, burns, and toothache	0.56	—	1/1.17	—	—	—
Colubridae								
*Philodryas olfersii* (Lichtenstein, 1823), Lichtenstein's green racer, cobra verde	Whole animal	Stroke	0.12	—	—	—	—	1/0.18
*Spilotes pullatus* (Linnaeus, 1758), yellow rat snake, caninana,	Bone and fat	Sore throat, cancer, and inflammations	0.35	—	—	—	—	1/0.42
Elapidae								
*Micrurus ibiboboca* (Merrem, 1820), caatinga coral snake, cobra-coral	Fat	Rheumatism, asthma, toothache, sore throat, cough, osteoporosis, swelling, inflammations, arthritis, and healing	0.57	—	—	3/0.97	—	—
*Caudisona durissa* (Linnaeus, 1758), rattlesnake, cascavel, LR	Fat, rattle, bone, and skin	Backache, rheumatism, cracks in the feet, osteoporosis, swelling, inflammation, arthritis, arthrosis, asthma, sore throat, earache, healing, burns, toothache, cough, bronchitis, snake bites, stroke, muscular pain, injuries, epilepsy, cancer, tuberculosis, “simpatias,” “evil eyes,” “attract partners,” and “attract money”	1.70	2/1.67	3/1.38	2/2.00	4/1.88	3/0.96
Unidentified family								
Alligator, jacaré_Aracaju	Skin	Stroke, asthma, bronchitis, and rheumatism	0.38	3/0.94	—	—	—	—
Alligator, jacaré_Maceió	Skin	Stroke, bronchitis, backache, toothache, snake bite, and rheumatism	0.53	—	—	2/0.98	—	—
Alligator, jacaré_Recife	Fat and skin	Sinusitis, bronchitis, asthma, sore throat, toothache, burns, thrombosis, cancer, and diarrhea	0.71	—	—	—	4/1.35	—
Alligator, jacaré_Salvador	Skin	Asthma, stroke, tuberculosis, sexual impotence, snake bites, inflammations, menstrual cramps, headache, and stomach ache	1.07	—	—	—	—	6/1.24

*Birds*								
Anatidae								
*Anser anser* (Linnaeus, 1758), greylag goose, ganso	Fat	Flu, cough and sore throat	0.18	—	—	—	1/0.28	—
*Anas platyrhynchos* Linnaeus, 1758, mallard, pato	Fat and eggs	Asthma, sore throat, sinusitis, and sexual impotence	0.30	—	—	—	—	2/0.54
Ciconiidae								
*Coragyps atratus* (Bechstein, 1793), black vulture, urubu, LR	Fat, liver, and e feather	“Simpatias,” alcoholism, and asthma	0.35	—	1/0.24	4/0.44	2/0.18	—
Columbidae								
*Columba livia* Gmelin, 1789, common pigeon, Pombo	Fat	Sore throat, sinusitis, cough, and asthma	0.21	—	—	—	—	2/0.26
Phasianidae								
*Gallus gallus*, (Linnaeus, 1758), chicken, galinha	Fat and spur	Cough, sore throat, flu, sinusitis, sexual impotence, swelling, nasal congestion, fever, diarrhea, earache, skin diseases, strain, burns, menstrual cramps, inflammations, pains, bruise, and cracks in the feet	1.33	2/0.89	2/0.55	—	2/0.34	4/1.62
*Numida meleagris* (Linnaeus, 1758), helmeted guineafowl, galinha d'angola	Fat	Nasal congestion, flu, rheumatism, asthma, strain, burns, and healing	0.48	—	—	—	—	1/0.58
*Pavo cristatus* (Linnaeus, 1758), common peafowl, pavão	Fat and feather	Snake bite, asthma, and sore throat	0.26	—	—	1/0.44	—	1/0.28
Rheidae								
*Rhea americana* (Linnaeus, 1758), greater rhea, ema, NT	Fat	Acne, rheumatism, cracks in the feet, burns, and nasal congestion	0.49	—	—	—	1/0.76	—
Unidentified family								
Owl, coruja_Fortaleza	Fat	“Simpatias”	0.12	—	1/0.24	—	—	—
Owl, coruja_Salvador	Fat	Sore throat, rheumatism, toothache, sinusitis, pains, and infections	0.44	—	—	—	—	1/0.54
Unidentified family								
Pheasant, faisão	Fat	Sore throat, rheumatism, toothache, sinusitis, and pains	0.41	—	—	—	—	1/0.50
Unidentified family								
Hawk, gavião	Fat	Asthma, cough, sore throat, fever, diarrhea, and earache	0.53	—	—	—	—	1/0.64

*Mammals*								
Agoutidae								
*Cuniculus paca* (Linnaeus, 1766), spotted paca, paca, LR	Penis	Sexual impotence	0.12	—	—	—	—	1/0.14
Bovidae								
*Bos taurus* (Linnaeus, 1758), cow, boi	Fat, tail, skin, horn, and fel (bile)	Asthma, sore throat, flu, stroke, bronchitis, thrombosis, swelling, diarrhea, sexual impotence, “evil eyes,” and “simpatias”	0.77	—	4/0.24	4/0.60	2/0.53	5/0.82
*Bubalus bubalis* (Linnaeus, 1758), water buffalo, buffalo	Tail and horn	Asthma, stroke, and “evil eyes”	0.35	—	—	—	1/0.41	—
*Capra hircus* (Linnaeus, 1758), domestic goat, bode	Fat and brain	Cracks in the feet, burns, sore throat, asthma, sinusitis, pain, toothache, rheumatism, osteoporosis, infections, inflammations, bruise, erysipelas, strain, and sexual impotence	0.98	1/0.56	—	2/1.04	1/0.76	2/0.72
*Ovis aries *(Linnaeus, 1758), sheep, carneiro	Fat	Rheumatism, cracks in the feet, sore throat, osteoporosis, arthritis, arthrosis, healing, inflammations, cough, swelling, flu, asthma, burns, pains, muscular pain, acne, circulatory problems, joint pain, bruise, strain, and toothache	1.09	2/1.11	5/1.31	3/1.36	12/1.57	1/0.46
Bradypodidae								
*Bradypus* sp., sloth, preguiça	Fat, nail, and skin	Thrombosis, stroke, asthma, sore throat, “evil eyes,” and circulatory problems	0,44	—	—	4/0.71	2/0.64	—
Canidae								
*Canis lupus* (Linnaeus, 1758), dog, cachorro	Fat	“Simpatias”	0.12	—	1/0.24	—	—	—
*Cerdocyon thous* (Linnaeus, 1766), fox, raposa, LR	Fat	Flu, asthma, pains, inflammations, snake bites, strain, rheumatism, and sore throat	0.59	—	—	4/1.09	1/0.18	—
Cervidae								
*Mazama gouazoubira* (G. Fischer, 1814), gray brocket, veado, VU	Tail, horne, and tíbia	“Simpatias,” asthma, sore throat, cough, arthritis, and rheumatism	0.44	—	2/0.93	3/1.20	4/0.95	2/0.48
Dasypodidae								
*Dasypus novemcinctus* (Linnaeus, 1758), nine-banded armadillo, tatu galinha, LR	Fat and tail	Deafness, earache, asthma, burns, sinusitis, cough, pains, inflammations, urinary infection, strain, and rheumatism	0.85	—	—	1/0.71	1/1.15	—
*Euphractus sexcinctus* (Linnaeus, 1758), six-banded armadillo, tatu-peba, LR	Fat, tail, and legs	Deafness, earache, asthma, burns, sinusitis, cough, pains, inflammations, strain, rheumatism, “evil eyes,” urinary infection, sexual impotence, injuries, tuberculosis, infections, and osteoporosis	1.22	—	—	1/0.71	1/1.20	3/1.22
Delphinidae								
*Sotalia guianensis *(P.-J. van Bénéden, 1864), Guianan river dolphin, boto, DD	Fat, penis, eyes, and blood	“Simpatias,” “attract partners,” asthma, rheumatism, osteoporosis, burns, sexual impotence, swelling, inflammations, arthrosis, toothache, headache, stomach ache, earache, erysipelas, flu, cough, sore throat, muscular pain, menstrual cramps, cancer, tuberculosis, pneumonia, thrombosis, snake bites, skin diseases, healing, and backache	1.90	1/1.22	1/0.24	7/1.58	6/1.79	6/1.58
Erethizontidae								
*Coendou prehensilis* (Linnaeus, 1758), Brazilian porcupine, porco-espinho, coandú, LR	Spine	Asthma, bronchitis, cough, thrombosis, cancer, eczema, acne, toothache, stroke, “attract money,” and earache	0.94	1/0.83	—	3/0.54	1/0.71	6/0.64
Felidae								
*Leopardus pardalis *(Linnaeus, 1758), ocelot, jaguatirica, gato maracajá, DD	Eyes	Asthma, “evil eyes,” and sexual impotence	0.35	—	—	—	1/0.53	—
Myrmecophagidae								
*Myrmecophaga tridactyla *(Linnaeus, 1758), Anteater, tamanduá bandeira	Skin	Stroke	0.12	—	—	—	—	1/0.14
Phyllostomidae								
*Desmodus rotundus* (E. Geoffroy, 1810), vampire bat, morcego, DD	Whole animal	Asthma, stroke, and rheumatism	0.35	—	—	—	—	3/0.42
Suidae								
*Sus scrofa* (Linnaeus, 1758), pig, porco	Fat	Acne, boils, and bronchitis	0.26	—	—	—	1/0.41	—
Trichechidae								
*Trichechus manatus* (Linnaeus, 1758), manatee, peixe-boi, CR	Fat	Burns, earache, swelling, wounds, menstrual cramps, bruises, asthma, flu, toothache, inflammations, pains, rheumatism, arthrosis, arthritis, backache, osteoporosis, cancer, thrombosis, sore throat, sinusitis, erysipelas, snake bites, skin diseases, healing, headache, cracks in the feet, and sexual impotence	1.87	1/1.22	—	4/1.63	2/1.28	3/2.00
Unidentified family								
Whale, baleia_Recife	Fat	Sore throat and menstrual cramps	0.23	—	—	—	1/0.36	—
Whale, baleia_Salvador	Fat	Rheumatism and menstrual cramps	0.23	—	—	—	—	2/0.28

Legends: RI: relative importance; CR: critically endangered; EN: endangered; VU: vulnerable; NT: near threatened; LR: lower risk; DD: deficient data.

**Table 2:** Categories of diseases treated with animal-based medicines that are sold in public markets in Aracaju-SE, Fortaleza-CE, Maceió-AL, Recife-PE, and Salvador-BA, according to the “Centro Brasileiro de Classificação de Doenças” (1993).

Categories	Diseases cited “by the vendors”	Total
A	“Attract money,” “attract partner,” “simpatias,” “evil eyes,” itch, bruise, pain, skin disease, edema, weakness, swelling, inflammations, infections, circulation problems, and lung problems	15
B	Asthma, bronchitis, nasal congestion, sore throat, flu, pneumonia, sinusitis, and cough	8
C	Arthritis, arthrosis, healing, backache, toothache, joint pain, osteoporosis, and rheumatism	8
D	Earache and deafness	2
E	Alcoholism, injuries, muscular pain, strain, snake bites, and burns	6
F	Stomach ache and gastritis	2
G	Acne, boils, eczema, and cracks in the feet	4
H	Sexual impotence	1
I	Stroke, thrombosis, and hemorrhoids	3
J	Urinary infection and menstrual cramps	2
K	Headache and epilepsy	2
L	Diarrhea, erysipelas, and tuberculosis	3
M	Cancer	1
N	Fever	1

Total		58

A: undefined illnesses; B: diseases of the respiratory system; C: diseases of the osteomuscular system and conjunctive tissue; D: diseases of the ear; E: lesions caused by poisoning and other external causes; F: diseases of the digestive system; G: diseases of the skin and the subcutaneous tissue; H: mental and behavioural perturbations; I: diseases of the circulatory system; J: diseases of the urogenital system; K: diseases of the nervous system; L: diseases caused by parasites; M: neoplasias (tumours); N: symptoms not categorized in other part or section.

**Table 3:** Consensus factors of the informants for the categories described.

	Categories													
	A	B	C	D	E	F	G	H	I	J	K	L	M	N
All localities combined														
Species used	38	56	29	20	22	11	19	9	22	8	6	17	11	2
Percentage of species used (%)	55.8	82.3	42.6	29.4	32.3	16.2	27.9	13.2	32.3	11.7	8.8	25	16.2	2.9
Use citations	259	613	269	51	107	25	42	22	98	17	20	30	20	2
Percentage of use citations (%)	16.4	38.9	17.1	3.2	6.8	1.6	2.6	1.4	6.2	1.1	1.3	1.9	1.3	0.12
ICF	0.88	0.91	0.89	0.62	0.8	0.58	0.56	0.61	0.78	0.56	0.73	0.44	0.47	—
Aracaju-SE														
Species used	10	12	9	4	3	3	2	1	5	—	—	—	—	—
Percentage of species used (%)	52.6	63.1	47.3	21	15.8	15.8	10.5	5.3	26.3	—	—	—	—	—
Use citations	27	45	28	5	8	4	3	1	13	—	—	—	—	—
Percentage of use citations (%)	20.1	33.6	20.9	3.7	5.9	3	2.2	0.7	9.7	—	—	—	—	—
ICF	0.65	0.75	0.7	0.25	0.71	0.33	0.5	—	0.66	—	—	—	—	—
Fortaleza-CE														
Species used	19	23	12	4	4	3	6	1	—	1	—	—	—	—
Percentage of species used (%)	67.8	82.1	42.8	14.3	14.3	10.8	21.4	3.6	—	3.6	—	—	—	—
Use citations	69	155	79	7	4	9	10	1	—	1	—	—	—	—
Percentage of use citations (%)	20.6	46.3	23.6	2	1.2	2.7	3	0.3	—	0.3	—	—	—	—
ICF	0.74	0.85	0.85	0.5	—	0.75	0.44	—	—	—	—	—	—	—
Maceió-AL														
Species used	14	26	12	7	6	2	6	1	8	3	1	4	1	—
Percentage of species used (%)	51.8	96.3	44.4	25.9	22.2	7.4	22.2	3.7	29.6	11.1	3.7	14.8	3.7	—
Use citations	59	150	98	17	20	4	11	4	24	5	6	5	2	—
Percentage of use citations (%)	14.5	37	24.1	4.2	5	0.9	2.7	0.9	5.9	1.2	1.5	1.2	0.4	—
ICF	0.78	0.83	0.88	0.62	0.73	0.66	0.5	1	0.69	0.5	1	0.25	1	—
Recife-PE														
Species used	17	24	12	9	9	2	7	3	8	3	—	1	2	—
Percentage of species used (%)	54.8	77.4	38.7	29	29	6.4	22.5	9.8	25.8	9.8	—	3.2	6.4	—
Use citations	59	136	30	14	47	3	14	4	13	4	—	5	3	—
Percentage of use citations (%)	17.7	40.9	9	4.2	14.1	0.9	4.2	1.2	3.9	1.2	—	1.5	0.9	—
ICF	0.72	0.83	0.62	0.39	0.82	0.5	0.54	0.33	0.41	0.33	—	0.75	0.5	—
Salvador-BA														
Species used	15	26	13	4	10	3	6	6	15	4	6	14	7	2
Percentage of species used (%)	41.6	72.2	36.1	11.1	27.7	8.3	16.6	16.6	41.6	11.1	16.6	38.8	19.4	5.5
Use citations	46	127	34	4	28	4	7	12	48	8	14	20	15	2
Percentage of use citations (%)	12.4	34.4	9.2	1.1	7.6	1.1	1.9	3.2	13	2.7	3.8	0.5	4.1	0.5
ICF	0.69	0.8	0.63	0.57	0.66	0.5	0.17	0.64	0.7	0.57	0.61	0.32	0.5	—

A: undefined illnesses; B: diseases of the respiratory system; C: diseases of the osteomuscular system and conjunctive tissue; D: diseases of the ear; E: lesions caused by poisoning and other external causes; F: diseases of the digestive system; G: diseases of the skin and the subcutaneous tissue; H: mental and behavioural perturbations; I: diseases of the circulatory system; J: diseases of the urogenital system; K: diseases of the nervous system; L: diseases caused by parasites; M: neoplasias (tumours); N: symptoms not categorized in other part or section.

**Table 4:** Comparison of observed species richness in Aracaju-SE, Fortaleza-CE, Maceió-AL, Recife-PE, and Salvador-BA public markets and the estimated species richness predicted by the estimators.

	Cities				
	Aracaju	Fortaleza	Maceió	Recife	Salvador
Sobs	19	28	27	31	36
Estimators					
ICE	25	34	28	49	48
Chao2	21	33	28	43	45
Jack1	25	36	29	44	49
Jack2	25	40	29	51	55

Sobs: observed species.
